# Overview and understanding of mental health and psychosocial support in Afghanistan

**DOI:** 10.1093/inthealth/ihad055

**Published:** 2023-07-25

**Authors:** Lyla Schwartz, Hannah Lane, Zainab Hassanpoor

**Affiliations:** Peace of Mind Association, 147 Wheel Meadow Drive, Longmeadow, Boston MA, 01106, United States; Peace of Mind Association, 147 Wheel Meadow Drive, Longmeadow, Boston MA, 01106, United States; Peace of Mind Association, 147 Wheel Meadow Drive, Longmeadow, Boston MA, 01106, United States

**Keywords:** Afghanistan, conflict, development, mental health, MHPSS

## Abstract

More than four decades of war in Afghanistan has been both a main driver for poor mental health, and a barrier to the development of crucial mental health services. A study conducted by BMC Psychiatry in 2021, across eight regions in Afghanistan, found staggering levels of depressive and anxiety disorders among the general population. Almost one-half of those interviewed (47.12%) reported having high levels of distress in the last month, and almost 40% (39.44%) reported experiencing impairment to their lives due to poor mental health. Yet, despite the common experiences of much of the population, mental health is a hugely stigmatized topic of discussion in Afghanistan, due to a myriad of cultural, religious, socioeconomic and environmental factors. And now, under the de-facto Taliban government, mental health has been deprioritized in the face of a crumbling economy and acute levels of poverty, all but forgotten. This paper sought to review the impact and change to mental health services under the de-facto government, and to provide the reader with greater awareness into the current situation in Afghanistan and equip them with insight into how to respond to the mental health needs of Afghans.

## Introduction

Mental health disorders disproportionately affect lower-middle income countries. It has been estimated that globally 29% of people with psychosis receive mental health treatment; in high-income countries that percentage is 70%, in low-income countries it is as low as 12%.^1^ It is common that mental health disorders are associated with, and exacerbate, inequalities. Factors such as unemployment and poverty increase the risk of developing mental health disorders, and often the most vulnerable groups are also the least supported, in terms of accessing healthcare and advocacy.^1^ Despite the high levels of poor mental health, treatment gaps continue to be high, with a scarcity of human resources, stigma, lack of prevention and promotion programs, as well as a lack of integration of mental health into primary care settings often being the cause.^2^ In addition, there is a lack of investment in mental health treatment in Afghanistan; the estimate for mental health is <4% of the overall health budget.^3^ A promising approach would be for national and international actors to provide additional investment in human resources and integrate mental health by ‘piggy backing’ on existing public health programs.^4^

In Afghanistan, much of the general population lives in a perpetual state of distress, exacerbated by a prolonged conflict that has ravaged the country for decades, and, as of late, unpredictable and volatile political instability. A 2018 European Union survey found that 85% of the Afghan population had experienced or witnessed at least one traumatic event in their life, with an average of four.^5^ For many, this trauma is internalized and minimized, increasing the likelihood of developing mental health conditions. Despite this, the mental health services across Afghanistan are insubstantial, and there is little funding for, or understanding of, mental health within the medical field. There is poor mental health literacy across Afghanistan, with much of the population finding it difficult to describe mental health distress and issues effectively. Without the appropriate language to reflect their experiences, Afghans are limited in the ways they can seek help.^6^

A report in 2021 found that <10% of the population utilizes any mental health service.^3^ And even then, those who do use them can experience abuse, such as forced hospitalizations,^3^ or being taken to religious shrines by family members where they may be tied and chained.^7^ Symptoms of mental illness are often categorized as a punishment from God, and harmful misconceptions mean mental illness is often considered a ‘weakness’ or defect in the person.^7^ In 2022, the WHO reported the prevalence of stigma around mental health resulting in those with mental disorders often being perceived as weak, lazy and unintelligent.^1^ These consequences extend beyond the individual and can affect the family unit, with the widely held belief that one person's ‘bad’ behavior can potentially lead to illness or even disability of a family member. Instead of being offered psych-medication or a form of psychotherapy, many of those with mental illness are instructed to pray for recovery, or are simply ostracized from the community.

There is a trend of misdiagnosing and overprescribing in Afghanistan, with patients presenting with psychosomatic conditions being prescribed medication, for example, for stomach issues, in place of being offered psychological support.^3^ Without appropriate support, Afghans may feel compelled to self-medicate for mental health disorders. Substance abuse is a common form of self-medication, with the reciprocal link with depressive disorders being well documented.^8^ Afghanistan has the highest number of opiate users globally, with an average in 2015 of 12.6% Afghan adults using drugs, compared with a lower global average of 5.2%.^8^ It is highly likely that this percentage has grown over the last 7 y.^8^ Substance abuse, particularly if it leads to addiction, only exacerbates poor mental health.

This combination of factors—high levels of trauma and instability, high levels of stigma and severely limited resources—has led to unprecedented and widespread mental illness in the Afghan population, and a mental health service ill equipped to respond. In this commentary paper, we provide an overview of mental health (MH) and mental health and psychosocial support (MHPSS) in Afghanistan, based on our findings through interviews with individuals who have worked in this sector—including a hospital director in Bamiyan, and a former member of the Ministry of Public Health (MoPH) in Afghanistan—all remaining anonymous for their own safety; in addition to our own experience in Afghanistan providing MHPSS services to communities and through consultancy services; supported by a comprehensive review of relevant mental health programming reports. A number of critical issues related to mental health including the accessibility afforded to different demographic groups, the significant increase in trauma experienced by women under the de-facto government and the deprioritization of MHPSS, are discussed. There exists a number of standardized approaches to MHPSS developed by international organizations such as the WHO; however, these approaches are not contextualized and therefore are not applicable in places like Afghanistan, where infrastructure and resources are severely lacking and trauma is chronic. By synthesizing our own knowledge and experience on the ground, with findings from the literature review and the primary data gathered from fellow mental health experts, we were able to identify in what ways the situation has changed since the de-facto government came to power. This paper seeks to summarize our findings and expertise for the benefit of international development actors and health practitioners; to provide insight and a broader understanding of the situation in Afghanistan, as well as to outline key approaches to implementing an effective mental health response under current circumstance.

## Mental health in Afghanistan through a demographic lens

The causes and rates of poor mental health in Afghanistan are not uniform. Different demographic groups are treated differently in society and are able to access support in different ways. Women in Afghanistan report higher levels of depression, anxiety and post-traumatic stress disorder (PTSD) than men, as a result of the heightened sociocultural stressors placed on them.^9^ A 2019 report found that >90% of suicides committed by women in Afghanistan are directly attributed to untreated mental illness,^7^ and a 2021 report found that 9.1% of women have reported having suicidal thoughts at one point in their life. While there is no one singular reason for this epidemic, one cause of developing a mental illness is abuse. Violence against women and girls is widespread and, according to a study by the United Nations Population Fund, 87% of Afghan women have been victims of at least one form of physical, sexual or psychological violence, and 62% have experienced multiple forms of abuse throughout their life.^10^ Alongside mental health stigma, there is significant stigma associated with abuse, leading many women to remain silent.

While women are likely to receive additional trauma, it is important that men are not neglected when it comes to mental healthcare. Young men in particular face challenges, and frequently stigma around seeking help is more acute among men than women, meaning they are less likely to come forward for support. This lack of support for men can indirectly affect women, particularly if unaddressed, as there is a significant correlation between trauma and domestic violence.^11^

Afghanistan in general has a youth bulge and economic hardship and lack of prosperity affects the younger generation more than any other group.^12^ Because of the conflict in Afghanistan, young people have lost decades of opportunity, in education, health and employment, as well as the opportunity to participate in politics. Armed non-state groups have profited from the vulnerability and disillusionment felt by youth, developing effective methods of recruitment and radicalization that the state is unequipped to deal with. As a result, children who have been radicalized and trafficked, transnationally or from within Afghanistan, often experience acute psychological trauma. But there is a significant lack of systematic psychological support services available to these children, especially with regard to effective interventions aimed at rehabilitation and reintegration of radicalized youth.

The Hazara people, an ethnic minority group who make up an estimated 15–20% of Afghanistan's population,^13^ is facing a mental health crisis since the Taliban government came into power in 2021. After a relatively peaceful 20 y since the previous Taliban government, the Hazara have once again come under attack for their religious and ethnic identity. In October 2021, a Shia mosque in southern Kandahar was attacked, killing at least 47 people and wounding 70 more.^13^ More recently, an attack on Abdulrahim Shahid High School in Kabul killed 30 and wounded scores of others.^13^ Furthermore, the group is heavily marginalized. For example, as the Taliban government took power, Hazara judges and judicial staff were removed from their positions, dismissed on the basis of not aligning with the administration's vision.^13^ With this onslaught of violent attacks, and the expulsion from political and social circles, thousands of Hazars are experiencing the effects of repeated trauma, an increase in paranoia, acute stress and depression.^14^

The quality and availability of mental health services also fluctuates regionally and negatively affects those living in rural settings to a greater extent. A WHO report from 2018 found that 87% of Afghanistan's population were within a 2-h distance of a health facility.^15^ However, more recently, in the north of Afghanistan, rural areas in Andrab, Panjshir and some parts of Balkhab are experiencing higher levels of violence and discrimination, which in turn impacts how healthcare operates, with mental healthcare practically non-existent. In other areas, including parts of Badakhshan, some rural communities reported having to travel for up to 9 h to receive even basic health treatment.

## The impact of regime change on mental health

The overthrow of the Islamic Republic of Afghanistan by the Taliban in August 2021 followed 3 mo of intensive conflict. Although data about the impact of these events on the mental health of the population are limited, one study of university students from August to November 2021 revealed incredibly high levels of trauma.^16^ Of the 217 students involved in the study, 70% reported experiencing significant PTSD symptoms, and 38.6% reported heightened suicide ideation. Although this was a very specific, highly educated and mostly female group of subjects, the findings illustrate how likely it is that the events of 2021 have contributed to the already high levels of trauma in the wider population. There are also increasing concerns about the ability of individuals to meet their basic needs and, in part due to the freezing of foreign reserves to prevent misuse by the Taliban, Afghans are increasingly facing poverty and starvation. According to The United Nations Development Program (UNDP), 97% of Afghans will fall into poverty this year. The World Food Programme also predicts that >20% of the population (8.7 million people) are at risk of famine-like conditions. These figures are stark, and legitimate fears about survival will be affecting many in the country. This widespread trauma and persistent political instability limits the ability of Afghans to live secure, fear-free lives. Anxieties about regime change and future violence, and the residual stress, lead to tangible impacts on mental health and quality of life.

In a 2019 survey by the Asia Foundation, only 13.4% of Afghans reported having sympathy or support for the Taliban.^19^ Alongside this, the Taliban does not have a history of understanding or prioritizing mental health. As a result, there is a lack of faith in the new government to continue funding health services to the same extent as their predecessors, or treat members of the population who are inhibited by mental illness with respect or dignity.

Under the current regime, there are well-founded fears about the stripping back of rights, particularly for women and girls, and the impact this could have—and is already having—on the population. Evidence of this occurring is already present, with a ban on girls’ secondary education being implemented in March 2022, and increasing limitations on the right of women to work. A report from early 2022 found that women make up to 75% of those who visit Herat's psychiatric hospital, with an increase of 7% in the number of mentally ill women receiving psychological treatment since a year prior,^18^ with most of the patients who were previously working or studying being treated for depression and anxiety.^18^ This hostile environment will be having significant impacts on mental health across the country, particularly on already vulnerable groups.

Widespread poor mental health impacts both the individual and society. Leaving mental illness untreated can lead to chronicity and the development of psychosomatic disorders, ultimately increasing suffering and the cost of care.^19^ At an individual level, as symptoms of mental illness intensify, quality of life is increasingly impacted. One's ability to engage with everyday activities is undermined and relationships often break down. In a country like Afghanistan where the ability to be productive and contribute to the family unit is vital, the implications of low productivity can be fatal. At a societal level, the greater percentage of the population that is struggling with poor mental health, the less productive and robust the economy is.

## Mental health response in Afghanistan

Mental health is seldom prioritized in humanitarian relief efforts, and currently only 2.1% of government health budgets globally are allocated to mental health.^20^ Recently there has been a shift in understanding, whereby mental health and psychosocial well-being is not only considered a health problem, but a social problem affecting all aspects of life, including peace and stability.^21^ The use of MHPSS in fragile and humanitarian contexts has increased and we are seeing the integration of mental health into wider health responses, and, less so, into wider social programming in protection or education sectors.^20^ MHPSS is a multifaceted and holistic approach to mental healthcare that not only treats the problem, but also works to build capacity of community-based responses, raise awareness of mental health and de-stigmatize the conversation, and equip people with coping skills and greater resilience; with the aim of reducing the likelihood of mental health disorders developing in the future. There is a growing appreciation for approaches to healthcare that are community based and contextualized to the culture, as well as the religious and familial structures of the beneficiaries. This is particularly relevant in Afghanistan, a widely patriarchal society with religion at the helm. Creating evidence-based approaches that apply to the population, in line with social values and cultural practices, not only address poor mental health but improve psychosocial well-being. Psychosocial support facilitates resilience, fosters dignity and independence, improves emotion regulation and promotes social cohesion and cooperation.^22^ It addresses both the psychological and social needs of people on an individual and community level.

However, despite the need for mental health reform in the country, MHPSS in Afghanistan remains scarce. The changeover of regimes only impeded any progress being made, and mental health has been further deprioritized with a devastating effect.

## MHPSS services prior to the changeover of governments

Soon after the previous Taliban government (1994–2001) was ousted and with the establishment of the Islamic Transition Government of Afghanistan in 2002, the new government developed 14 developmental programs within its National Development Framework. One such program was formed by the MoPH, the Consultative Group on Health and Nutrition, a group of national and international actors put together to inform health-related policy and development. And thus, the Basic Package of Health Services (BPHS) was designed, a core package of essential health services developed to address priority health needs and make basic healthcare accessible to all. At the time, maternal mortality rates and under-five mortality rates were dire, and a particular focus of the UNDP.^23^ Geographical access was also in focus, with only 10% of the population living within a 1-h walking distance of a health facility.^24^ It was not until the expansion of the strategy in 2005 that mental health was designated as a top priority.^25^ However, given the wider challenges of delivering on healthcare, issues such as maternal and child health, nutrition and communicable diseases all remained a higher priority.^26^

Prior to the change of governments in 2021, the BPHS had been contracted out to non-governmental organizations (NGOs) across 31 provinces. Levels in Afghanistan's healthcare system were distinct, and outpatient care and community support were provided through six different types of facilities:^27^

- **Health post**

Basic healthcare delivered in its simplest form, and can be as humble as a room in someone's house dispensing aspirin.

- **Basic health centers (BHCs)**

A small center frequently set up in an existing building that has been transformed and rarely newly built, as NGOs rarely have capital to build infrastructure.

- **Mobile health teams (MHTs)**

Teams of healthcare professionals that go into communities, and even family homes, to provide care. Particularly used in emergencies or to serve hard-to-reach or rural populations.

- **Comprehensive health centers (CHCs)**

Similar to a BHC, but has the capacity for an overnight stay; and a CHC Plus has the capacity for slightly longer stays.

- **District hospitals**

Small to medium hospitals situated across each province.

- **Provincial hospitals**

Larger hospitals with greater capacity, frequently situated in provincial capital cities.

The BPHS was designed to be scaled up rapidly by contracting work out to NGOs; yet the contracts were flawed and breach of contract was common. For example, BPHS contracts did not require infrastructure changes of any kind, such as a wall around the compound, which for many ‘makeshift’ centers meant a lack of privacy. In Afghan culture, modesty is regarded as highly important, and so families were unlikely to send members, in particular women, to seek medical care at such a place.

Care was not standardized across Afghanistan, despite the uniformity of BPHS requirements, because there was a lack of consequences for those organizations that did not meet contractual obligations. The BPHS would dictate the number of skilled staff and service lines each kind of clinic needed. For example, a BHC may be required to have one manager, one administrative staff member, one doctor, one nurse, one laboratory assistant and so forth, and would be provided an Essential Medicine List, where it might say, the center must have rabies shots and pain medication available. However, there was often a dearth of certain qualified practitioners, and in some cases centers would claim they were unable to find anyone to fill vacancies in order to save on budgets. The MoPH could do nothing when centers failed to recruit, as the lack of qualified medical practitioners was common knowledge. Furthermore, the requirement for a mental health professional was very uncommon. Those trained in mental health will often work for NGOs as opposed to hospitals or other clinical settings. This is, at least in part, due to NGOs offering a substantially higher salary than the state can provide.

The MoPH had a shepherding role over the NGOs providing healthcare in 32 of the country's provinces—with everything overseen by the ministry—despite it having little part in implementing care. In 2002, when decisions were being made on how the new republic would operate, an overwhelming amount of unmet need was identified. There were gaps in every sector, even in farming and livestock management, despite Afghanistan being an agrarian society. Thus, the government outsourced health to NGOs, with the U.S. Agency for International Development (USAID) playing a significant role in this process, and divided primary care from secondary and tertiary care. Primary care would be provided through the BPHS, divided by catchment; each province would govern the levels of healthcare in this vicinity. Few new structures were built; instead, healthcare was delivered in existing, often unfit, infrastructure.

In 32 of 34 provinces in Afghanistan, the BPHS was operational, and implemented by NGOs. The remaining two provinces, Kabul and Kapisa, received direct government healthcare, operating under USAID's Health Sector Resiliency project. Kapisa was kept under government provision with the intent of using this province as an example to exemplify the ineffectiveness of government-delivered healthcare. However, this ploy was not successful, and the province in fact proved to operate just as well as the provinces under NGO care. This was due to the unique situation of its staffing. Kapisa's healthcare workforce were people who were from the province themselves, which fostered a ‘local sense of ownership’ among healthcare workers and recipients.

There are few mental health institutions in Afghanistan, and these are scattered across the country with limited capacity and low levels of coverage. Afghanistan has a total of two psychiatric hospitals in the country, one in Kabul and one in Herat. The Red Crescent Secure Psychiatric Institution, a high security psychiatric hospital in Herat, houses >300 patients, some of whom are at times chained and sedated.^28^ A report from 2018 on the hospital found patients were often overstaying, due to a severe lack of adequate outpatient mental health services.^28^ Often, people will go directly to hospitals to seek help, and bypass smaller health centers, knowing they have greater capacity and are more likely to have appropriate medicine and trained doctors. However, this in turn overwhelms the hospitals.

## MHPSS services since the changeover of governments

Mental health services are currently operating under the previous government's system, headed by the Department of Mental Health within the MoPH. These services were already deprived of capacity, quality and access under the previous government, but with the deprioritization of mental health this has further deteriorated and the MoPH at present is struggling to be an effective coordinating body. Stigma has remained a main barrier to receiving care, and the everyday situational risks of living in Afghanistan have meant mental health is no longer in focus. This risks creating a system where mental health will not be understood, advocated for, integrated or culturally competent.

Over the past two decades the financing of health systems in Afghanistan was largely supported by international donors such as the WHO and USAID; however when the Taliban took power international donors immediately suspended most of their funding. This funding constituted >70% of government expenditure, according to a report by The International Committee of the Red Cross (ICRC), including many health facilities.^29^ A significant contribution to this was the Sehatmandi project, with a US$600 million budget; administered by the World Bank and implemented by the MoPH, this project was designed to increase the utilization and quality of healthcare by supporting the delivery of BPHS across all 34 provinces.^30^ This was a 3-y project that ran from June 2018 until June 2021, with many Afghans in the health sector hoping for renewal. But, with the withdrawal of major international funding under the Taliban government, this has not materialized. A report from September 2021 found that only 17% of the >2300 health clinics under the Sehatmandi project were fully functioning.^31^ With the lack of funding, many clinics previously operating under the Sehatmandi project have not been able to pay staff and are struggling to maintain sufficient supplies of medicines, including psychotropic drugs.^15^ As a result, Afghans are forced to travel further for essential medical care, to self-medicate, or to go without.

A report by the MoPH from 2017 found that while primary healthcare in the country is perceived to be dependent on international donors, in reality, a large proportion of total health spending is carried by households.^32^ Now, with soaring prices and inflation, an estimated 17 million Afghans (in a population of roughly 38 million) are experiencing serious food insecurity, and, compounded by the looming famine in many parts of the country, healthcare, and in particular mental healthcare, is viewed as a non-essential cost for many Afghans.^33^

Since the changeover of government, the Afghan people have become more desperate for psychological support. Organizations such as HealthNet TPO have continued to deliver mental healthcare through MHTs, prescribing medicines and providing counseling.^34^ And others, such as CARE, have psychosocial counselors on staff when responding to emergencies, such as the recent earthquakes in Paktika and Khost.^35^ However, mental health has been deprioritized since the Taliban government came into power, and health concerns such as malnutrition take priority.

## Recommendations

People in Afghanistan are widely exposed to adversity, whether it be from individual circumstances or national crises. MHPSS is about empowering people to be able to respond to difficult circumstances, cope with adversity and build resilience; improving their overall quality of life. Negative emotions linked to circumstances someone is not in control of, such as conflict or disaster, only perpetuate symptoms of poor mental health. And so, promoting a future-oriented perspective is key to improving mental health. Short of major political and medical reform in the country, mental health needs to be prioritized by the international NGO community, to see progress and recovery for those struggling with persistent mental health disorders. There are several key approaches NGOs and the medical community can take to better respond to mental health needs in Afghanistan.

1. **Strengthening the system**

To effectively address the mental health needs of Afghans, it is imperative that a framework of family-focused and community-based care is established and supported. Afghanistan is a low-resource country and providing stand-alone clinical mental health treatment is not sustainable in the long term. Developing and implementing a framework in which social, public health and clinical services are delivered in tandem at a community level can help to fill the gaps in previous models of healthcare, in particular improving coordination of care, addressing the comorbidity of physical and psychological conditions, and lessening the need for outside referrals. Furthermore, in Afghanistan, where family is the most important social unit of society, family-focused care would greatly improve accessibility for the most vulnerable members of a community. For example, a woman's mental health is highly affected by that of her husband's, and so, by treating the major issues of the head of the family (typically the husband or father), the health of the remaining members of the family can be positively affected. Likewise, in treating a child, the health needs of the primary carer—often the mother—must also be treated.

2. **Advocacy for change: hearing from the community**

Raising awareness and advocating for mental health through a bottom-up approach should be a high priority. Advocacy is most successful when the target community is asked to become part of the solution and encouraged to participate in deciding how mental health support is delivered, in a way that is socially and culturally acceptable to its members.

3. **Leading by example: the role of national professionals**

A promising approach would be to utilize the influence of leadership in the medical field. It is unrealistic to try and de-stigmatize a population without adjusting the attitudes and beliefs of the medical force serving them. Afghanistan is a heavily community-driven collectivist society, with great importance placed on the opinions of community leaders. De-stigmatizing and normalizing discussions around mental health are essential for large-scale recovery to occur, and health professionals can take on a major role in changing the narrative.

4. **Working alongside the government**

The current government is focused on security, hunger and other crises, with mental health not viewed as a priority, and an absence of capacity or incentive to change this is apparent. If the government cannot meet the need for mental health services independently, it has to be subsidized externally. NGOs and international INGOs should work with health practitioners on the ground to deliver MHPSS, under government oversight.

5. **Building the capacity building of health practitioners**

Investing in people and services (rather than physical infrastructure), training and providing ongoing supervision to health practitioners is a realistic way of reaching greater numbers of Afghans in need. Large hospitals or health centers are not always accessible, physically or socially, and so building the capacity of rural health practitioners, those working with women, and those with access to minority communities, is important.

## Conclusion

Since the regime change in August 2021, mental health has been deprioritized on both an individual and a governmental level. Despite this, the need for contextualized mental health support remains high as conflict-related trauma is intensified by restrictions imposed by the de-facto government and the poverty that has ensued from the withdrawal of international support. Mental health facilities and practitioners remain few and far between, despite the greater numbers of people, in particular women, seeking psychological support. The withdrawal of funding from international sources has halted potentially significant programs from materializing; however, it is difficult to predict to what extent these programs would have impacted the masses, as previous health programs failed to fully integrate mental health into the public health response. With the de-facto government far removed from the mental health needs of the Afghan people, it is up to international NGOs to step in and support in building the capacity of existing systems so as to create sustainable change in the mental health response.

## Data Availability

The data underlying this article cannot be shared publicly due to the privacy and safety of respondents who provided first-hand experiences and opinions on the health sector in Afghanistan. Partial data may be shared on reasonable request to the corresponding author.
